# Novel “red‐bull sign” during cavotricuspid isthmus ablation: Indication of an ablation catheter stuck in the subeustachian pouch

**DOI:** 10.1002/joa3.12793

**Published:** 2022-11-07

**Authors:** Moyuru Hirata, Koichi Nagashima, Ryuta Watanabe, Yuji Wakamatsu, Naoto Otsuka, Satoshi Hayashida, Shu Hirata, Masanaru Sawada, Sayaka Kurokawa, Yasuo Okumura

**Affiliations:** ^1^ Division of Cardiology, Department of Medicine Nihon University School of Medicine Tokyo Japan

**Keywords:** atrial flutter, cavotricuspid isthmus, second‐generation irrigated catheter, subeustachian pouch, temperature‐controlled ablation

## Abstract

**Background:**

A subeustachian pouch (SEP) often hinders the completion of a cavotricuspid isthmus (CTI) ablation of typical atrial flutter (AFL) and sometimes causes steam‐pops during a power‐controlled ablation. We hypothesized that real‐time bull's‐eye monitoring of the catheter surface temperature might be useful to locate the SEP where the temperature can rise rapidly, and a temperature‐controlled ablation might avoid steam pops. This study aimed to demonstrate this hypothesis.

**Methods:**

A temperature‐controlled CTI ablation with a QDOT MICRO™ catheter (*n* = 10) and a conventional power‐controlled CTI ablation (*n* = 10) were performed with an output power of 35 W. During the RF application, the bull's eye monitor for monitoring the catheter surface temperatures was assessed. A “red‐bull sign” was defined as an entire red‐colored bull's‐eye monitor, indicating that the catheter‐tip temperature of all 6 thermocouples rose rapidly over 47°C.

**Results:**

In a total of 115 lesions (12 ± 3 per patient), a “red‐bull sign” was observed in 39 (33.9%) lesions where the RF output was reduced to 26 ± 8 W. All 39 “red‐bull sign” lesions corresponded to the location of the SEP as delineated by ICE before the ablation. The red‐bull sign accurately indicated the presence of a SEP with a sensitivity of 84.7% and specificity of 100%. Bidirectional block of the CTI was completed in all patients in either catheter group without any steam‐pops.

**Conclusion:**

Real‐time surface temperature monitoring and a red‐bull sign might be useful to detect the SEP. A temperature‐controlled CTI ablation with the QDOT MICRO catheter might be safe for avoiding steam pops.

## Graphical abstract

Real‐time catheter‐surface temperature monitoring with 6 thermocouples situated at the ablating electrode tip of the QDOT MICROTM catheter is useful for locating subeustachian pouches, which often hinder the completion of a cavotricuspid isthmus ablation of typical atrial flutter. A “red‐bull sign”, defined as the bull’s‐eye monitor colored entirely in red, indicating that the temperature on all 6 thermocouples rose rapidly over 47°C, accurately indicated the presence of a subeustachian pouch with a sensitivity of 84.7% and specificity of 100%.

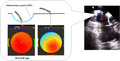

## 1 BACKGROUND

Radiofrequency (RF) ablation of the cavotricuspid isthmus (CTI) is a highly successful procedure for typical atrial flutters (AFLs), and bidirectional block of the CTI is a commonly determined endpoint.^1^ The combined use of intracardiac echocardiography (ICE) and a 3D mapping system is useful to delineate the CTI anatomy including a subeustachian pouch (SEP) and the Eustachian ridge.^2^ However, the SEP often hinders the completion of bidirectional block of the CTI and sometimes causes steam‐pops during a power‐controlled RF application with an irrigated ablation catheter.^3^ Therefore, the management of the RF applications within the SEP is a key to complete the CTI line without any complications.

The recent development of a second‐generation irrigated catheter (QDOT MICRO™ catheter, Biosense Webster) equipped with 6 thermocouples situated at the surface of the electrode tip has rejuvenated the potential utility of the temperature‐controlled ablation with an automatic adjustment of the irrigation flow and RF power output, reducing the risk of steam pops and collateral damage.^4^, ^5^ However, the utility of the real‐time temperature assessment during the CTI ablation has not been clarified. We hypothesized that the real‐time monitoring of the catheter surface temperature might be useful to locate the SEP where the temperature can rapidly rise, and a temperature‐controlled ablation might avoid steam‐pops.

## 2 METHODS

### 2.1 Study design

This retrospective study included 20 patients with AFL who underwent a CTI ablation with the combined use of ICE and a 3D mapping system at Nihon University Itabashi Hospital: 10 patients with a QDOT MICRO™ catheter (QDOT group; between August 2021 and September 2021 during an external evaluation period of the QDOT MICRO™ catheter prior to the pre‐commercial use in Japan) and 10 with a conventional irrigated tip catheter (ThermoCool SmartTouch™, Biosense Webster) (STSF group; between November 2018 and September 2022). The study was approved by the Institutional Review Board of Nihon University Itabashi Hospital, and an opt out system was used to obtain the patients' content for the use of their clinical data for research purposes.

### 2.2 Electrophysiologic study

Under conscious sedation achieved with dexmedetomidine and fentanyl, an electrophysiologic study and PVI were performed with the use of a 3‐dimensional mapping system (CARTO3, Biosense Webster), steerable sheath visualized on the CARTO3 system (VIZIGO, Biosense Webster), and ICE catheter (SOUNDSTAR, Biosense Webster). A multielectrode catheter (BeeAT, Japan‐Life‐Line) was placed in the coronary sinus (CS) through the right subclavian vein and another multielectrode catheter (Electrode: 1 mm, 2‐5‐2 mm interelectrode spacing; POPLALYON™ Boston Scientific) was placed adjacent to the tricuspid annulus through the femoral vein. The 12‐lead electrograms and bipolar intracardiac electrograms were recorded with a band‐pass filter setting of 30–500 Hz at a paper speed of 100–200 mm/s and stored on a digital recording system (LabSystem PRO, Bard Electrophysiology).

### 2.3 Anatomical visualization of the cavotricuspid isthmus and ablation

**FIGURE 1:**
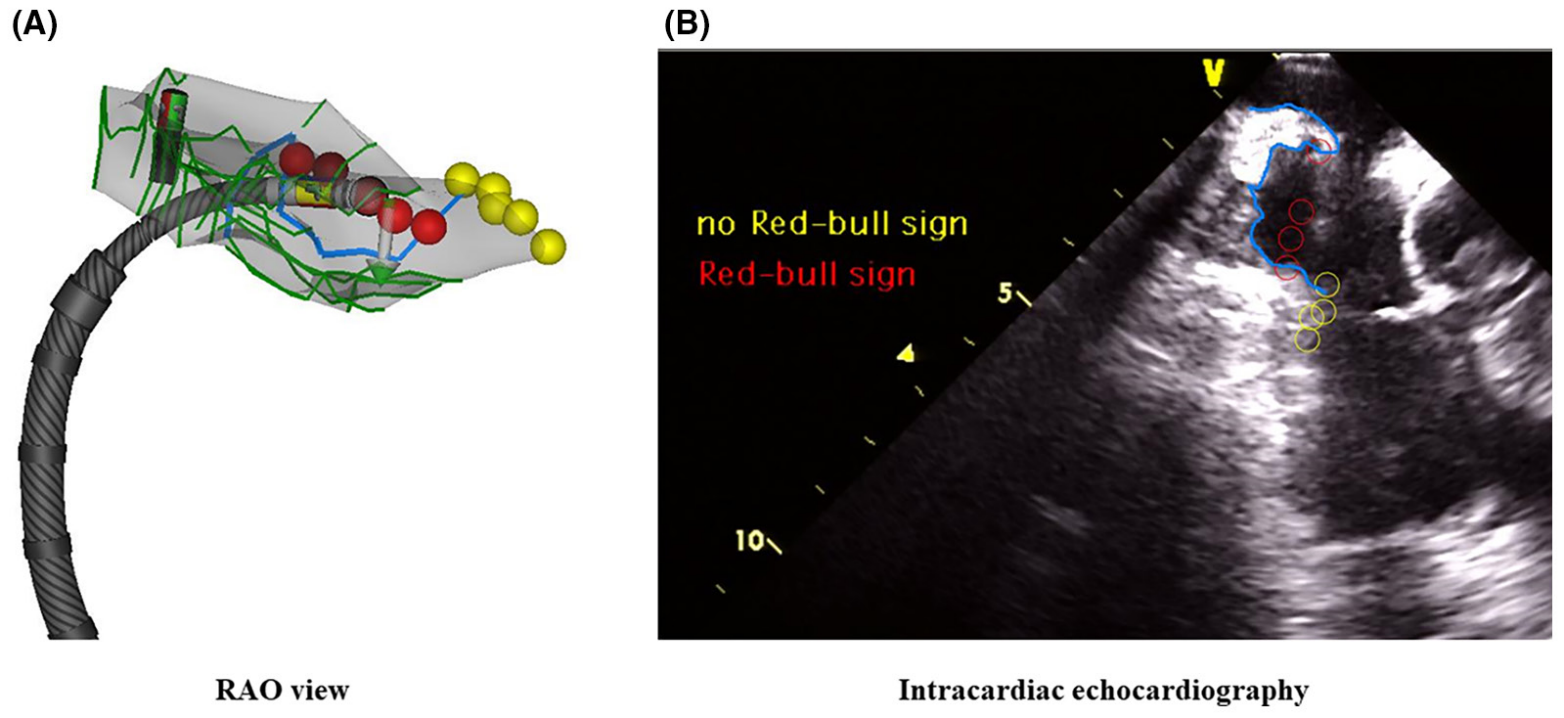
A representative example of the radiofrequency application in the subeustachian pouch (SEP) of cavotricuspid isthmus (A), which is delineated by the intracardiac echocardiography (B). The SEP is delineated as a blue line. A red‐bull sign is observed in all lesions in the SEP. RAO, right anterior oblique.

**FIGURE 2:**
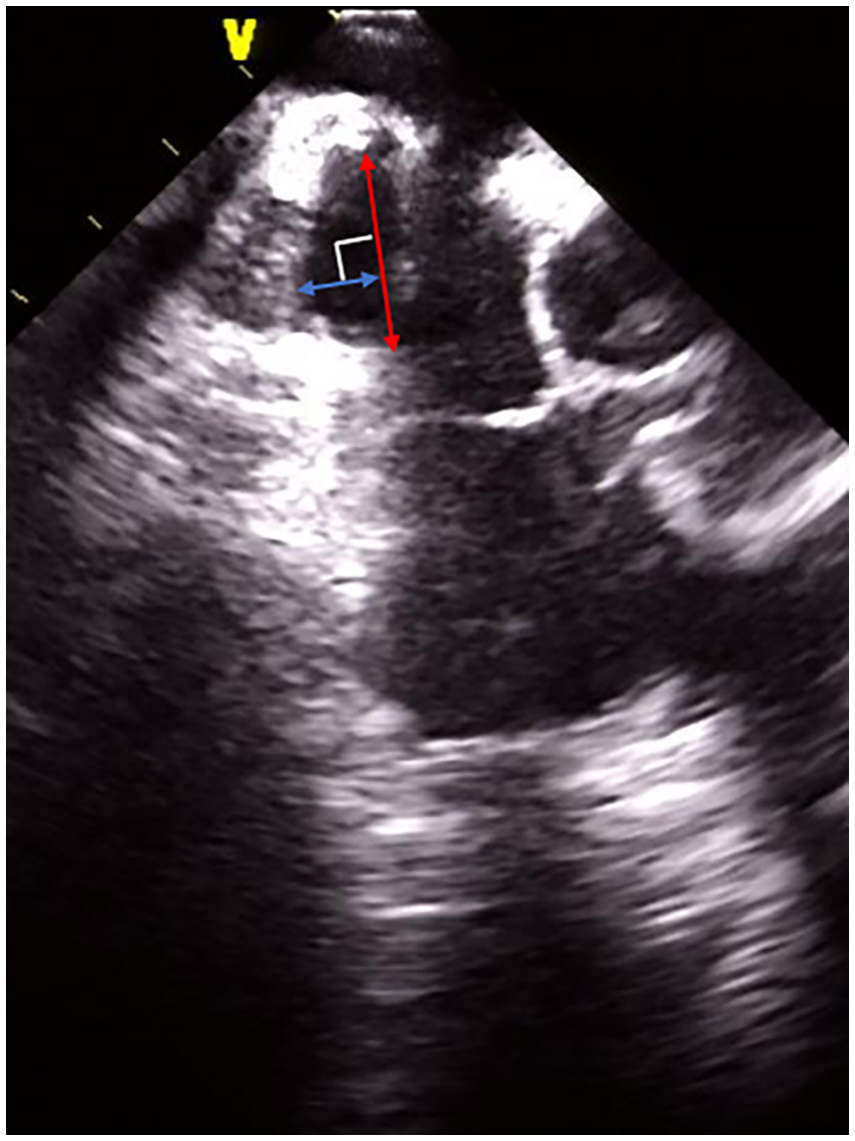
A representative example of the maximal diameter and depth of the subeustachian pouch (SEP). The red line and blue line indicate the diameter and depth, respectively. SEP, subeustachian pouch.

After the PVI, the geometry of the CTI was created and the SEP was delineated by the ICE (Figure 1). We performed a CTI ablation with a point‐by‐point RF delivery during either AFL or pacing from the proximal CS electrode, using the geometry of the CTI. From a view where the maximal longitudinal diameter of the SEP was observed during the P wave onset, that is, the maximal volume of the RA, the SEP‐related variables were measured as follows: the maximal diameter defined as the length of an imaginary line connecting the tops of the edges of the SEP and maximal depth measured as the length of a vertical line connecting the deepest site and SEP diameter line (Figure 2). “The SEP edge area” was defined as the area within 4 mm of the SEP edge, which was arbitrarily determined according to the VISITAG diameter. “Inside the SEP” was defined as the area inside the SEP except for the SEP edge area.

**FIGURE 3:**
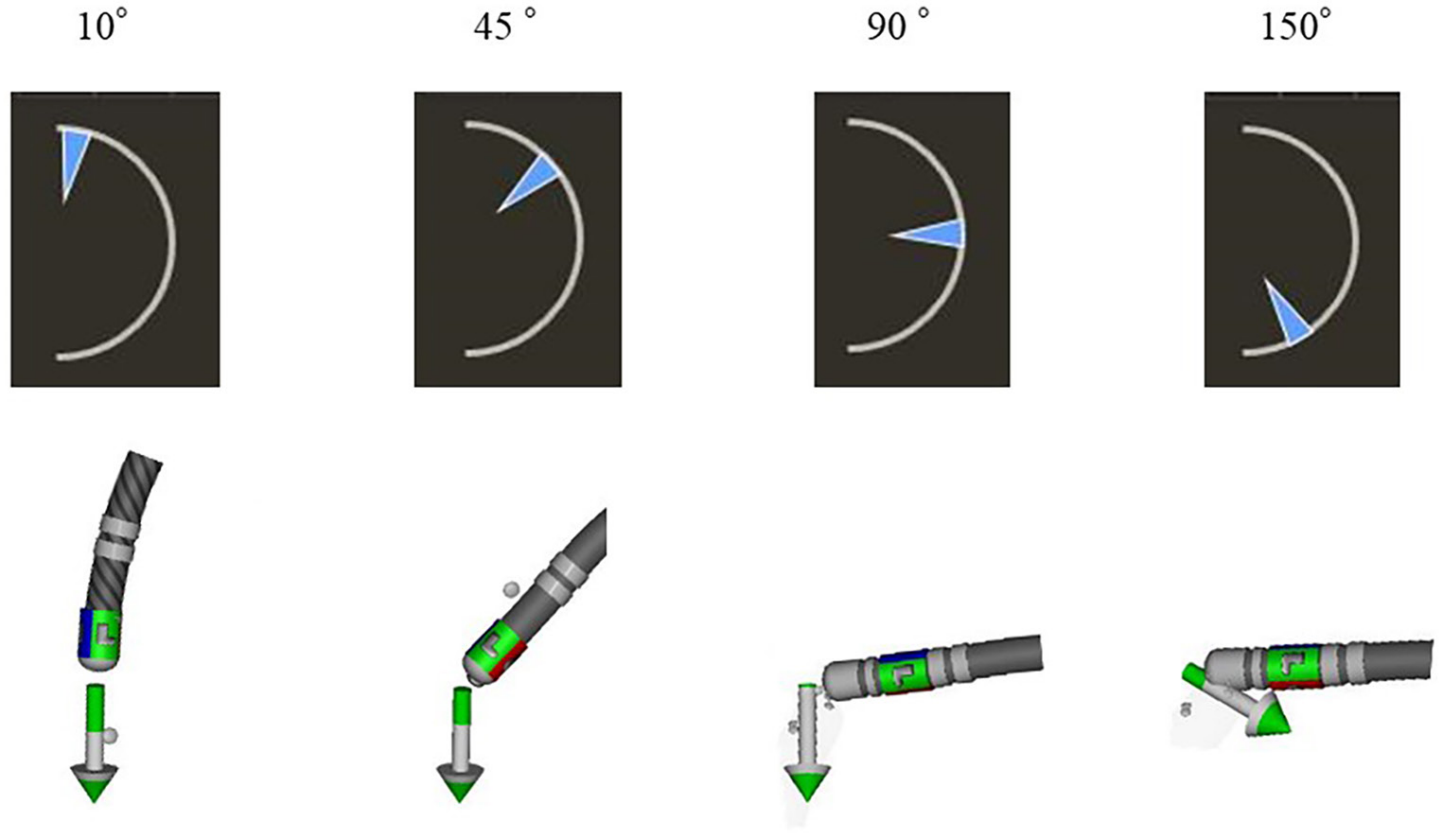
Representative examples of an angle between the contact vector of the ablation catheter and catheter shaft.

Temperature‐controlled ablation was performed with the QDOT MICRO™ catheter and a radiofrequency generator (nGEN RF Generator, Biosense Webster) with an initial output power of 35 W and irrigation flow of 4 ml/min. A contact force of 10–20 g was targeted and the RF delivery time for the lesion was set to 30 s. The real‐time catheter‐surface temperature assessed by the 6 thermocouples situated on the surface of the ablating electrode tip was sent to the generator every 33 ms during the RF application and visualized in the bull's eye monitor. The mode of the temperature‐controlled ablation was the QMODE (≤35 W). If the real‐time surface temperature increased to over the target temperature, which was set at 47°C as a previous report,^5^ the irrigation flow automatically increased to 4–15 ml/min; if the temperature was still over the target temperature with the irrigation flow at 15 ml/min, the power output of the RF automatically decreased until the temperature reached the target temperature. A “red‐bull sign” was defined as the bull's‐eye monitor colored entirely in red, indicating that the temperature on all 6 thermocouples rose rapidly over 47°C (Movie S1). The “red‐bull sign” was assessed within the SEP and outside the SEP. When a red‐bull sign was observed, the RF delivery within the SEP was continuously performed and the output power was immediately automatically reduced. A conventional power‐controlled CTI ablation was performed with an STSF with similar settings such as an output power of 35 W, target contact force of 10–20 g, and 30 s RF duration. The ablation‐related parameters potentially related to the catheter surface temperature were the average contact force (CF), ablation index (AI), and contact vector of the ablation catheter for each lesion. A representative example of the angle measured between the contact vector of the ablation catheter and ablation catheter shaft is shown in Figure 3. The completion of the bidirectional block of the CTI was confirmed by differential pacing with an output of 10 mA and pulse width of 2 ms from the low lateral right atrium and CS.

### 2.4 Statistical analysis

The continuous variables are expressed as the mean ± SD values. Categorical variables are expressed as the number and percentage of patients. A Student's *t*‐test was used to analyze the differences in the continuous variables, and a Fisher's exact test was used to analyze the differences in the dichotomous variables. A *p* value of <.05 was considered to be statistically significant. All statistical analyses were performed with JMP 16 software (SAS Institute).

## 3 RESULTS

### 3.1 Patient characteristics and CTI ablation features between the QDOT and STSF groups

The patient characteristics and CTI ablation features are shown in Table 1. The patient characteristics were similar between the 2 groups. The SEP was delineated with ICE in 17/20 patients (85%) (9/10 in the QDOT group and 8/10 in the STSF group [*p* = .53]). There were no differences in the SEP features including the maximal diameter and depth. The ablation‐related variables were similar between the 2 groups, such as the number of RF lesions, total RF delivery time, first‐pass CTI completion rate, and mean AI. However, the average contact force and angle between the contact vector and ablation catheter shaft was higher in the QDOT group than STSF group possibly due to the difference in the period when either catheter was used. The SEP features in each patient are shown in Table 2.

**TABLE 1:** Patient and CTI ablation characteristics (*n* = 20)

	QDOT group (*n* = 10)	STSF group (*n* = 10)	*p* value
Patient characteristics			
Age (years)	70 ± 10	69 ± 13	0.62
Male sex	6 (60%)	8 (80%)	0.32
BMI (kg/m^2^)	23 ± 3	22 ± 3	0.32
LVEF (%)	72 ± 8	70 ± 4	0.48
LAD (mm)	39 ± 6	37 ± 6	0.52
CHA_2_DS_2_‐VASc score	2.0 ± 1.1	1.8 ± 1.1	0.70
Clinical arrhythmia			0.10
Paroxysmal AF	7 (70%)	8 (80%)	
Persistent AF	3 (30%)	0	
AFL	0	2 (20%)	
CTI ablation			
Total			
First‐pass CTI completion	6 (60%)	5 (50%)	0.53
Average CF (g)	15 ± 5	12 ± 4	<0.0001
AI	479 ± 56	488 ± 38	0.13
Angle between the contact vector and ablation catheter shaft	80 ± 52°	66 ± 39°	0.012
Total RF lesions (per patient)	12 ± 3	17 ± 9	0.11
Total RF delivery time (min)	9.9 ± 5.0	17.0 ± 12.9	0.12
Within the SEP			
Presence of an SEP	9 (90%)	8 (80%)	0.53
Maximal SEP depth (mm)	9 ± 3	6 ± 3	0.05
Maximal SEP length (mm)	13 ± 6	14 ± 5	0.74
Average CF (g)	15.9 ± 5.1	12.3 ± 4.3	0.0008
AI	472 ± 56	472 ± 44	0.94
Angle between the contact vector and ablation catheter shaft	63 ± 47	58 ± 40	0.62
Total RF lesions (per patient)	4 ± 3	5 ± 3	0.60
Total RF delivery time (min)	2.3 ± 1.9	1.9 ± 1.4	0.52
Outside the SEP			
Average CF (g)	15.9 ± 5.1	12.3 ± 4.3	<0.0001
AI	485 ± 55	494 ± 34	0.14
Angle between the contact vector and ablation catheter shaft	91 ± 52	68 ± 39	0.009
Total RF lesions (per patient)	7 ± 2	13 ± 2	0.05
Total RF delivery time (min)	2.9 ± 1.4	5.6 ± 3.6	0.04

*Note*: The mean ± SD values or number (%) of patients are shown.

Abbreviations: AF, atrial fibrillation; AFL, atrial flutter; AI, ablation index; BMI, body mass index; CF, contact force; CTI, cavotricuspid isthmus; LAD, left atrial diameter; LVEF, left ventricular ejection fraction; RF, radiofrequency; SEP, subeustachian pouch.

**TABLE 2:** The characteristics of the SEPs

QDOT group			STSF group	
Patient	Maximal SEP diameter (mm)	Maximal SEP depth (mm)	Maximal SEP diameter (mm)	Maximal SEP depth (mm)
1	4.3	7.1	12.7	3.8
2	8.0	12.0	21.8	11.2
3	8.6	13.3	no SEP	
4	no SEP		no SEP	
5	14.9	7.2	21.0	8.5
6	19.6	13.1	12.6	5.6
7	9.9	4.2	14.7	6.5
8	11.5	6.2	11.7	4.8
9	19.7	7.7	8.5	3.9
10	20.2	10.5	7.6	3.8

*Note*: The maximal diameter and depth of the subeustachian pouch are shown for every case.

Abbreviation: SEP, subeustachian pouch.

Out of a total of 115 RF lesions (12 ± 3 lesions per patient) in the QDOT group, a red‐bull sign was observed in 39 (33.9%). The RF output automatically decreased down to 26 ± 8 W in those lesions, resulting in a lower AI as compared to the lesions without a red‐bull sign (463 ± 53 vs. 487 ± 55, *p* = .03). The CF also tended to be lower in the lesions with the red‐bull sign than in those without (14.2 ± 4.6 g vs. 16.2 ± 5.5 g, *p* = .06). Within the SEP, there were no differences in the AI, angle between the contact vector and ablation catheter shaft, total RF lesions, and total RF delivery time between the QDOT and STSF groups. Outside the SEP, there were no differences in the AI between the QDOT and STSF groups. However, the catheter angle was higher in the QDOT group than STSF group. The total RF delivery time was lower in the QDOT group than STSF group (2.9 ± 1.4 vs. 5.6 ± 3.6 min, *p* = .04). However, this difference was mainly reflected in the RF lesions and time outside the SEP. Bidirectional block of the CTI was achieved in all cases with/without additional RF deliveries adjacent to the SEP. The total RF delivery time was 9.9 ± 5.0 min. No steam pops occurred in either group.

### 3.2 “Red‐bull sign” as a predictor of a SEP ablation

The association of lesions within or outside the SEP with the presence of a red‐bull sign is shown in Table 3. All lesions with a red‐bull sign corresponded to the SEP location as delineated by the ICE, regardless of different maximal SEP diameters and depths. The red‐bull sign accurately indicated the presence of a SEP with a high diagnostic performance (sensitivity = 84.7%, specificity = 100%, Table 3). Segmentally, a red‐bull sign was observed in all lesions inside the SEP (sensitivity = 100%, specificity = 100%, Table 3), but it existed less prevalently at the SEP edge area (12/19 [63%] lesions).

**TABLE 3:** The assessment of the red bull sign

	The number of lesions within an SEP		The number of lesions outside the SEP	Total number of lesions
	Inside the SEP	The SEP edge area		
The number of lesions where a red‐bull sign was observed	27	12	0	39
The number of lesions where a red‐bull sign was not observed	0	7	69	76
Total number of lesions	27	19	69	115

*Note*: The number of lesions where the red‐bull sign or no red‐bull sign was observed.

Abbreviation: SEP, subeustachian pouch.

## 4 DISCUSSION

### 4.1 Main findings

The main findings of this study were as follows: (1) a “red‐bull sign” was observed during the RF delivery in SEPs with a diameter of ≤20.2 mm and depth of ≤13.3 mm (sensitivity: 84.7%, specificity: 100%), and the red‐bull signs always occurred inside the SEP without exception and less prevalently occurred at the SEP edge area (63%), (2) the RF power output automatically decreased down to 26 ± 8 W within the SEP despite the lower CF and AI, (3) the CTI ablation was successful in all patients in both the QDOT and STSF groups, and (4) no steam‐pops occurred in either group but there was a higher CF in the QDOT group.

### 4.2 Utility of the “red‐bull sign” for the CTI ablation

A SEP was detected by ICE in 85% of the patients in our study, which was a relatively higher prevalence as compared to a previous report.^6^ A previous study reported the utility of the delineation of the SEP by ICE and a 3D mapping system for an effective CTI ablation avoiding RF deliveries in the SEP and steam‐pops.^2^ However, the delineation of the CTI is time‐consuming and requires an expert technique of ICE use, which sometimes fails to detect the SEP.^2^ If a power‐controlled RF is delivered in the undetectable SEP, there is a risk for steam pops, which sometimes cause right atrial perforations.^7^, ^8^ Therefore, a reliable modality able to detect the SEP and provide a safety‐net such as a tissue‐temperature controlled ablation in an undetectable SEP is warranted. From our data, bull's eye temperature monitoring of the QDOT catheter would be useful for the detection of a SEP with a high sensitivity and specificity, and a temperature‐controlled CTI ablation with the QDOT MICRO catheter may also enable avoiding steam pops even in an undetected SEP. All lesions where a red‐bull sign was not observed were located at the SEP edge area. Given that, an RF delivery inside the SEP had a higher risk for steam pops rather than the SEP edge. No steam pops occurred despite the higher CF in the QDOT group possibly due to an automated setting for increasing the irrigation flow and to reduce the RF energy rapidly.^5^

The concern, however, would be the lesion durability in the SEP. In fact, the majority of the recurrence sites on the CTI line were located near the SEP although the number of RF lesion and RF time in the SEP was similar between the QDOT and STSF groups.^9^ Given that, the temperature‐controlled CTI ablation with the QDOT MICRO catheter might be safer than a conventional power‐controlled CTI ablation, and additional RF applications using the QDOT MICRO catheter adjacent to the SEP with a “red‐bull sign” might be crucial for preventing any future recurrences of the CTI ablation.

### 4.3 Limitations

This report was a retrospective study with a small population. A red‐bull sign was not observed outside the SEP in our study but might also be observed during RF deliveries between relatively large pectinate muscles.^10^ Furthermore, a red‐bull sign was not observed in 7 lesions at the SEP edge area. Therefore, during the RF deliveries at the SEP edge area where a red‐bull sign was not observed, we could not confirm whether or not the temperature within the SEP would entirely rise. The cutoff value for the SEP size for predicting a red‐bull sign was not determined in this small sample size. Further, the long‐term durability of the CTI ablation was not assessed. A larger study in a prospective fashion would be warranted.

## 5 CONCLUSION

Real‐time surface temperature monitoring and a red‐bull sign might be useful for locating the SEP.

## FUNDING INFORMATION

None.

## CONFLICT OF INTEREST

The authors have no financial conflicts of interest to disclosure.

## DECLARATIONS

*Approval of the research protocol*: The study was approved by the Institutional Review Board of Nihon University Itabashi Hospital, and an opt out system was used to obtain the patients' consent for the use of their clinical data for research purposes. *Informed consent*: N/A. *Registry and the Registration No. of the study/trial*: N/A. *Animal study*: N/A.

## Supporting information


Movie S1
